# Dr. Charles P. Clark

**Published:** 1913-10

**Authors:** 


Dr. Charles P. Clark, Toronto, 1889, for many years a practitioner of Buffalo, died suddenly while in his automobile, August 28, of heart disease. He was about 50 years of age. He was at one time a member of the faculty of Niagara University, and belonged to the University Club. He apparently had a momentary warning, as he had shut off the engine, but the machine ran a short distance with no guidance from the hand on the wheel.

